# Erythropoietin Levels Are Not Independently Associated with Malaria-Attributable Severe Disease in Mozambican Children

**DOI:** 10.1371/journal.pone.0024090

**Published:** 2011-08-30

**Authors:** Núria Díez-Padrisa, Ruth Aguilar, Sonia Machevo, Luis Morais, Tacilta Nhampossa, Cristina O’Callaghan-Gordo, Delino Nhalungo, Clara Menéndez, Anna Roca, Pedro L. Alonso, Quique Bassat

**Affiliations:** 1 Centre de Recerca en Salut Internacional de Barcelona, Hospital Clínic, Universitat de Barcelona, Barcelona, Spain; 2 Centro de Investigaçaõ em Saúde da Manhiça, Manhiça, Mozambique; 3 CIBER de Epidemiología y Salud Pública (CIBERESP), Barcelona, Spain; 4 Faculdade de Medicina, Universidade Eduardo Mondlane, Maputo, Mozambique; 5 Instituto Nacional de Saúde, Ministerio de Saúde, Maputo, Mozambique; Menzies School of Health Research, Australia

## Abstract

**Background:**

Severe malaria is difficult to differentiate from other forms of malaria or other infections with similar symptoms. Any parameter associated to malaria-attributable severe disease could help to improve severe malaria diagnosis.

**Methodology:**

This study assessed the relation between erythropoietin (EPO) and malaria-attributable severe disease in an area of Mozambique with moderate malaria transmission. 211 children <5 years, recruited at Manhiça District Hospital or in the surrounding villages, were included in one of the following groups: severe malaria (SM, n = 44), hospital malaria without severity (HM, n = 49), uncomplicated malaria (UM, n = 47), invasive bacterial infection without malaria parasites (IBI, n = 39) and healthy community controls (C, n = 32). Malaria was diagnosed by microscopy and IBI by blood/cerebrospinal fluid culture.

**Principal Findings:**

Mean EPO concentration in the control group was 20.95 U/l (SD = 2.96 U/l). Values in this group were lower when compared to each of the clinical groups (p = 0.026 C versus UM, p<0.001 C vs HM, p<0.001 C vs SM and p<0.001 C vs IBI). In the 3 malaria groups, values increased with severity [mean = 40.82 U/l (SD = 4.07 U/l), 125.91 U/l (SD = 4.99U/l) and 320.87 U/l (SD = 5.91U/l) for UM, HM and SM, respectively, p<0.001]. The IBI group [mean = 101.75 U/l (SD = 4.12 U/l)] presented lower values than the SM one (p = 0.002). In spite of the differences, values overlapped between study groups and EPO levels were only associated to hemoglobin. Hemoglobin means of the clinical groups were 93.98 g/dl (SD = 14.77 g/dl) for UM, 75.96 g/dl (SD = 16.48 g/dl) for HM, 64.34 g/dl (SD = 22.99 g/dl) for SM and 75.67 g/dl (SD = 16.58 g/dl) for IBI.

**Conclusions:**

Although EPO levels increase according to malaria severity and are higher in severe malaria than in bacteremia, the utility of EPO to distinguish malaria-attributable severe disease is limited due to the overlap of values between the study groups and the main role of hemoglobin in the expression of EPO.

## Introduction

An optimal management of malaria needs a correct diagnosis and a good assessment of severity. Presence of *Plasmodium* in blood can easily be determined by microscopy or rapid diagnostic tests (RDTs), but even when *Plasmodium* is observed, attribution of symptomatology to malaria is not straightforward. In areas that are hyper- or holo-endemic for malaria, children are exposed to repeated infective bites during infancy and early childhood, progressively acquiring immunity against the disease and developing clinical tolerance to malaria parasites [1]. Thus, the detection of parasites in the blood of sick patients is not a definitive proof of their association to the clinical symptoms. Furthermore, clinical signs of malaria in children are highly unspecific and often overlap with those of other infections such as pneumonia, bacteremia or meningitis [1]–[5], all major causes of morbidity and death among young children in developing countries [6]. In such a context, severe malaria is difficult to differentiate from other infections. Any parameter associated to malaria-attributable severe disease could help to improve severe malaria diagnosis [7].

Additionally, the broad diversity of pathophysiological pathways, leading to the different syndromic presentations associated to severe malaria, further hinders diagnosis. Severe malaria in African children encompasses at least three main clinical syndromes that can occur alone or in combination: severe malarial anemia, cerebral malaria and respiratory distress (often secondary to metabolic acidosis [8]). Acidosis, predominantly due to lactic acid accumulation, has emerged as a central feature of severe malaria [9]. Malarial acidosis is multifactorial [10], but results mostly from increased host anaerobic glycolysis due to a mismatch between tissue oxygen supply and requirement. Reduced tissue oxygenation is central in malaria pathophysiology [11], [12].

One of the mechanisms suggested to be causing tissue hypoxia in malaria is severe anemia, a frequent clinical syndrome secondary to hemolysis and suppression of erythropoiesis [13], [14]. Acute malaria hemolysis is characterized by increased levels of unconjugated bilirubin and lactate dehydrogenase (LDH). With the destruction of red blood cells, hemoglobin (Hb) and LDH are released into the circulation. Liberated Hb is converted into unconjugated bilirubin in the reticuloendothelial cells of the spleen and transported to the liver, where it is conjugated to glucoronic acid. However, when hemolysis is also extravascular, plasmatic levels of unconjugated bilirubin increase because hepatocytes cannot process the excess bilirubin [15]. The other two mechanisms suggested to be causing tissue hypoxia in malaria are obstruction of tissular blood flow, due to the adhesion of parasitized and poorly deformable non-parasitized red blood cells to endothelial walls of small vessels [16]–[18], and inhibition of mitochondrial function by cytokines and nitric oxide [19].

Erythropoietin (EPO), a glycoprotein hormone principally produced by the kidney’s peritubular capillary endothelial cells in response to hypoxia, is crucial for sustained proliferation and differentiation of erythroid cells [20]. Regulation of EPO is believed to rely on a feed-back mechanism measuring blood oxygenation through hypoxia inducible transcription factors [21]. In addition, EPO is bound by circulating red blood cells; low circulating numbers lead to a relatively high level of unbound EPO which stimulates their production in the bone marrow [22]. Novel pathways involving EPO indicate that the increased presence of this molecule during periods of oxidative stress may result in cellular mechanisms designed to protect from damaging reactive oxygen species [23]–[25]. Several studies have described high levels of EPO in African children with malaria [26]–[30], suggesting that EPO might be a good indicator of malaria-attributable disease.

Assuming that hypoxia-related acidosis is the cardinal sign of severe malaria and that EPO is the key molecule in the adaptation to hypoxia; we hypothesized that EPO levels could be related to malaria-attributable severe disease among children with different degrees of malaria severity and children with no-malarial causes of severe disease (bacteremia), in an area of Mozambique with moderate malaria transmission.

## Methods

### Study area and population

This study was conducted at the *Centro de Investigação em Saúde da Manhiça* (CISM) and the Manhiça District Hospital (MDH), the referral health facility for Manhiça District, a rural malaria-endemic area of Southern Mozambique.

Since 1996, CISM has been running a continuous Demographic Surveillance System (DSS) and a morbidity surveillance platform at the MDH. The DSS covers 500 km^2^ and approximately 80,000 inhabitants (18% children <5 years) [31]. Under-five mortality rate in the area was 139/1,000 in 2005 [31].

Malaria transmission, perennial but with substantial seasonality, is mainly due to *P. falciparum* [32]. In 2003–2005, malaria accounted for 30.5% of all pediatric outpatient visits [32] and 49% of all pediatric admissions [33]. Almost 19% of all in-hospital pediatric deaths were due to malaria [33]. According to WHO criteria, 13.2% of admissions had severe malaria, being prostration (55.0%), respiratory distress (41.1%) and severe anemia (17.3%) the 3 most prevalent clinical presentations [33].

In Manhiça, *P. falciparum* and anemia show a considerable overlap in the spatial and temporal distribution patterns. In a community cross-sectional survey among healthy children, the prevalence of anemia at the end of the rainy season was twice as high than at the end of the dry season (34.4% versus 17.9%), suggesting that malaria is an important contributor to anemia [34]. Additionally, a negative correlation between asexual *P. falciparum* density and packed cell volume was found [34]. Data collected in 2003–2005 confirmed this relation, showing an anemia prevalence of 47% among outpatient children <5 years, with 68.4% of the malaria cases being anemic [32].

Between 2001 and 2006, community-acquired bacteremia accounted for 8% of all pediatric admissions and 21% of all in-hospital deaths [35]. In 2006, acute bacterial meningitis was suspected in 18% of all pediatric admissions and confirmed in 7% of these cases, 24% of which died [36]. Severe malnutrition prevalence in children <5 years old admitted to MDH was 10% in 2001–2003 [37]. HIV prevalence among pregnant women attending the antenatal clinic was 23.6% in 2004 [38], mother-to-child transmission occurred in 12.4% of the births and HIV prevalence among the newborns was 3% [39].

### Study design

Children included in this analysis were recruited as part of a larger study designed to explore the use of certain cytokines/other proteins to differentiate malaria, bacterial and viral infections among children <5 years old seen at MDH. Recruitment of the larger study went from September 2006 to May 2009 and included, among other patients, children with different degrees of malaria severity or invasive bacterial infection. Data presented here belong to those children from the larger study fulfilling the group definitions of the present analysis and with plasma samples available to perform EPO measurements. Written informed consent was obtained from all participant parents/legal guardians involved in the study. The study was approved by the Mozambican National Bioethics Committee and Institutional Review Board of the *Hospital Clínic de Barcelona.*

Children included in this analysis were classified into five groups: severe malaria (SM), hospital malaria (HM), uncomplicated malaria (UM), invasive bacterial infection (IBI) and healthy control (C). SM group was further stratified in severe malarial anemia (SMA), cerebral malaria (CM) and mixed severe malaria (MSM) subgroups. We used the HM group to describe an intermediate severity group in malaria, including those children judged to need hospitalization but without the criteria needed to fulfill the severe malaria case definition. This non-standard classification, also used in previous malaria vaccine trials [40], may help classifying malaria cases in a gradient of increasing severity. Children from the clinical groups were recruited at MDH. For the control group, healthy children from the DSS area were randomly selected maintaining the same pattern of age in the clinical groups (40% children <1 year, 30% children 1 to <2 years and 30% children 2 to <5 years).

Standardized questionnaires with demographic and clinical data were completed for recruited children together with the following samples: i) finger prick blood smears for malaria microscopy; ii) 1–3 ml of venous blood for bacterial culture; iii) 1.5 ml of venous blood in EDTA for full blood count, EPO, bilirubin and LDH measurements. Blood culture was not performed for the control group. Cerebrospinal fluid (CSF) was only collected from hospitalized children with suspicion of meningitis. Children in the inpatient groups residing in the DSS area were offered voluntary HIV counseling and testing. Additional written informed consent and finger prick were needed for this purpose.

### Study groups

Severe malaria (SM) was defined as the presence of fever (axillary temperature ≥37.5°C) or reported history of fever in the preceding 24 hours in children admitted with *P. falciparum* in blood (≥500 asexual parasites/µl), a negative blood and CSF culture (if available) and, at least, one of the following criteria: severe anemia (hematocrit <15% or Hb <5g/dl), deep coma (Blantyre coma score ≤2), prostration (inability to sit unaided or look for mother’s breast/feed in children who cannot yet sit), hypoglycemia (<2.2 mmol/l), repeated convulsions (≥2 reported episodes in the 24 hours prior to admission) or respiratory distress (deep breathing or indrawing). Among SM group, children with only severe anemia were classified in the severe malarial anemia (SMA) subgroup and those with only neurological impairment including prostration, convulsions or/and coma in the cerebral malaria (CM) one. Finally, mixed severe malaria (MSM) subgroup included those SM cases with SMA or/and CM accompanied with any other criteria of SM and those SM cases with hypoglycemia or/and respiratory distress.

Hospital malaria (HM) was defined as the presence of fever or history of fever in the preceding 24 hours in children admitted with *P. falciparum* in blood (≥500 asexual parasites/µl), a negative blood and CSF culture (if available) and not fulfilling inclusion criteria for severe malaria.

Uncomplicated malaria (UM) was defined as the presence of fever or history of fever in the preceding 24 hours, *P. falciparum* in blood (≥500 asexual parasites/µl) and a negative blood culture in children seen as outpatients with no clinical criteria for hospital admission.

Invasive bacterial infection (IBI) was defined in children admitted with respiratory distress (chest indrawing and/or deep breathing) as the presence of bacteria isolated from blood and/or CSF and no *P. falciparum* in blood. Potential contaminants (*Staphylococcus epidermidis*, gram-positive *Bacillus* and, only in blood, *Streptococcus viridans*) were not included in this group.

Controls (C) included healthy children from the community with no history of fever or detectable fever at recruitment, no report of having received medical assistance/treatment during the last 30 days, no signs or symptoms of illness and no *P. falciparum* parasites in blood.

### Laboratory methods

*P. falciparum* parasites were detected by microscope observation of thick and thin Giemsa-stained blood films as described elsewhere [32]. Blood cultures were performed by incubating 1–3 ml of blood during 4 days using an automated system (BACTEC® 9050, Becton-Dickinson, Franklin Lake, NJ, USA). Positive blood cultures were examined following standard procedures [37], [41]. CSF were cultured using conventional methods, bacterial isolates were identified by colony morphologic analysis and growth requirements [36].

Full blood counts were performed using a *Sysmex KX21* hematology analyzer (Sysmex Long Grove, IL, USA). Plasma was obtained by blood centrifugation (5 minutes, 1500 rpm). Plasma LDH and total-bilirubin (conjugated plus unconjugated) were measured using a *Vitros DT60 II* biochemical analyser (Orthoclinical Diagnostics, Rochester, NY, USA). Remaining plasma was stored at −20°C and sent to the *Hospital Clínic de Barcelona*, where EPO quantification was performed using *Quantikine human Erythropoietin* (R&D Systems, Minneapolis, MN, USA) immunoanalysis with a limit of detection of 0.6 U/ml.

HIV testing was done using two RDT: *Determine* (Abbott Laboratories, North Chicago, IL, USA) and *Unigold* (Trinity Biotech, Bray, Ireland). For children <18 months positive by both RDT and for those cases of discordant results between RDT, HIV-1 infection was confirmed using antigen DNA-PCR Roche *HIV-1 DNA test* (Roche Molecular Systems, Branchburg, NJ, USA).

### Data management and statistical analysis

Data were double entered using *Fox Pro* version 2.6 (Microsoft Corporation, Redmond, WA, USA) and discrepancies between two entries were resolved referring to the original forms. Statistical analyses were performed using *STATA* version 11 (Stata Corporation, College Station, TX, USA).

Proportions were compared using Chi-square test. EPO and bilirubin values were logarithmically transformed to perform the analysis. In the case of malaria groups, test for trend was used to assess lineal increase of different parameters with severity. ANOVA test was performed to evaluate differences among distributions of EPO, hemoglobin, bilirubin and LDH levels between the study groups. Linear regression models were estimated to evaluate the difference of EPO levels adjusted by age, sex, malnutrition, hemoglobin and days of fever prior to visit/admission. Univariate linear regression was used to explore the relation between EPO/bilirubin/LDH and Hb. P-values ≤0.05 were considered significant.

## Results

### Study groups profile

This analysis is based on 211 children <5 years recruited between September 2006 and May 2009, 132 of which were admitted to hospital. Distribution of the children according to study groups was: SM (n = 44), HM (n = 49), UM (n = 47), IBI (n = 39) and C (n = 32). Children in the SM group presented at least one of the following criteria: respiratory distress (n = 29), severe anemia (n = 16), prostration (n = 14), convulsions (n = 9), deep coma (n = 2) and hypoglycemia (n = 1). Among these children, 8 corresponded to the SMA subgroup, 6 to the CM and 30 to the MSM. The following bacteria were isolated in the IBI group: *Streptococcus pneumoniae* (n = 22), *Haemophilus influenzae B* (n = 6), *Escherichia coli* (n = 4) *Staphylococcus aureus* (n = 3), *Salmonella* spp (n = 2), *Streptococus* spp (n = 1) and *Klebsiella pneumoniae* (n = 1). No bacterial co-infection was found.

Nine of the 124 admitted children with outcome data died in-hospital. All fatalities occurred in the IBI group, which had an associated case fatality rate of 28% (9/32). Severe anemia was more prevalent in the SM group when compared to the other groups (36% versus 5%, 0% and 0% in the IBI, HM and UM, respectively). Demographic and clinical features of the study groups are shown in Table 1.

**Table 1:** Demographic and clinical features of the study groups.

Variables	Group^a^					p-value	
	C(n = 32)	UM(n = 47)	HM(n = 49)	SM(n = 44)	IBI(n = 39)	HM,UM & SM^b^	SM & IBI
Age, months (n = 211)	17 (6–26)	31 (19–42)	22 (16–33)	25 (14–40)	10 (4–16)	0.211	<0.001
Sex (n = 211)							
Male	22 (69)	30 (64)	25 (51)	27 (61)	24 (62)	0.792	0.987
Female	10 (31)	17 (36)	24 (49)	17 (39)	15 (38)		
Days of fever prior to visit/admission (n = 162)							
≤3	NA^c^	44 (94)	37 (86)	30 (91)	32 (82)	0.605	0.279
>3	NA	3 (6)	6 (14)	3 (9)	7 (18)		
Severe malnutrition^d^ (n = 159)							
No	NA	46 (100)	39 (93)	33 (89)	26 (76)	0.032	0.153
Yes	NA	0 (0)	3 (7)	4 (11)	8 (24)		
Hemoglogin, g/dl (n = 173)	NA	93.98 (14.77)	75.96 (16.48)	64.34 (22.99)	75.67 (16.58)	<0.001	0.013
Severe anemia^e^ (n = 179)							
No	NA	47 (100)	49 (100)	28 (64)	37 (95)	<0.001	0.001
Yes	NA	0 (0)	0 (0)	16 (36)	2 (5)		
Lactic acidosis^f^ (n = 67)							
No	NA	NA	NA	18 (64)	29 (74)	NA	0.374
Yes	NA	NA	NA	10 (36)	10 (26)		
Oxigen saturation^g^ (n = 67)							
≥94%	NA	NA	NA	21 (75)	20 (49)		
90-<94%	NA	NA	NA	3 (11)	8 (19)	NA	0.145
<90%	NA	NA	NA	4 (14)	11 (32)		
HIV (n = 43)							
Negative	NA	NA	NA	15 (83)	10 (40)	NA	0.004
Positive	NA	NA	NA	3 (17)	15 (60)		
Outcome (n = 124)							
Alive	NA	NA	48 (100)	44 (100)	23 (72)	NA	<0.001
Death	NA	NA	0 (0)	0 (0)	9 (28)		

NOTE. Median and inter-quartile range (IQR) for age. Mean and standard deviation (SD) for hemoglobin. Other data are n (%) of patients.

a C: control, UM: uncomplicated malaria, HM: hospital malaria, SM: severe malaria, IBI: invasive bacterial infection.

b Test for linear trend.

c NA: Not applicable.

d Weight for age z-score <-3 from U.S. reference population.

e Hematocrit <15% or hemoglobin <5 g/dl.

f Plasma lactate >5 mmol/l.

### EPO levels in the study groups

**Figure 1:**
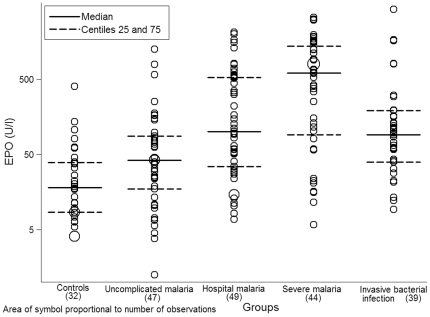
Distribution of EPO levels (U/l) in the study groups.

Although values overlapped between study groups, differences in the distribution of EPO levels were observed (p<0.001) (Figure 1). Mean EPO concentration in the control group was 20.95 U/l (SD = 2.96 U/l). Values in this group were lower when compared to each of the clinical groups (p = 0.026 C vs UM, p<0.001 C vs HM, p<0.001 C vs SM and p<0.001 C vs IBI). EPO levels in the 3 malaria groups increased with increasing severity (n = 47, 49 and 44, p<0.001), with mean values of 40.82 U/l (SD = 4.07 U/l) for UM, 125.91 U/l (SD = 4.99 U/l) for HM and 320.87 U/l (SD = 5.91 U/l) for SM. For the IBI group, mean EPO concentration was 101.75 U/l (SD = 4.12 U/l). Values in this group were lower than those in the SM group (n = 39 and n = 44, p = 0.002).

**Figure 2:**
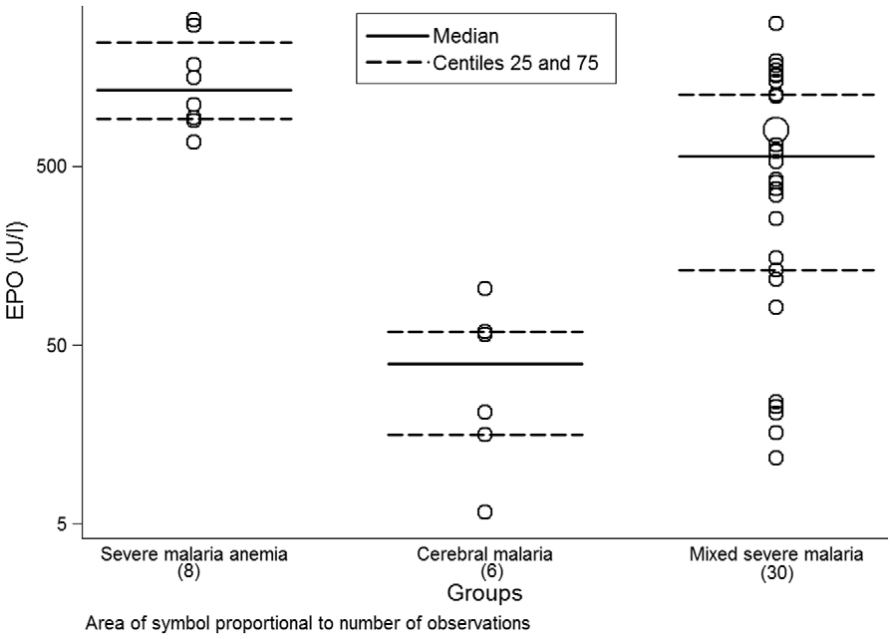
Distribution of EPO levels (U/l) in the SM subgroups.

When stratifying SM group in SMA, CM and MSM, differences on EPO levels were observed among the subgroups (n = 8, n = 6 and n = 30, p<0.001) (Figure 2), with mean values of 1439.38 U/l (SD = 1.79 U/l) for SMA, 29.45 U/l (SD = 2.89 U/l) for CM and 346.69 U/l (SD = 5 U/l) for MSM.

### Association of EPO levels with descriptive parameters in the clinical groups

The association of EPO levels with demographic and clinical parameters in the clinical groups is illustrated in Table 2. SM group was used as the baseline when comparing study groups. In the crude analysis, EPO levels appeared associated to the study group (n = 179, p<0.004 SM vs HM, p<0.001 SM vs UM and p = 0.001 SM vs IBI) and the Hb concentration (n = 173, p<0.001). An inverse relationship was observed between EPO and Hb [β(SE) = 0.93 (0.003), p<0.001], being Hb levels lower in the groups with higher levels of EPO. Hb levels decreased in the malaria groups (n = 42, 48 and 44, p<0.001), with mean values of 93.98 g/dl (SD = 14.77 g/dl) for UM, 75.96 g/dl (SD = 16.48 g/dl) for HM and 64.34 g/dl (SD = 22.99 g/dl) for SM. For the IBI group, mean Hb concentration was 75.67 g/dl (SD = 16.58 g/dl) and higher than in the SM group (n = 39 and 44, p = 0.013) (Table 1).

**Table 2:** Association of EPO levels with descriptive parameters in the clinical groups.

Variables	Crude analysis			Adjusted analysis a (n = 149)		
	Proportional difference	95% CI	p-value	Proportionaldifference	95% CI	p-value
Group (n = 179)						
SM	1			1		
HM	0.39	0.21–0.74	0.004	0.92	0.57–1.49	0.730
UM	0.13	0.07–0.24	<0.001	1.18	0.68–2.03	0.550
IBI	0.32	0.16–0.62	0.001	0.68	0.39–1.18	0.169
Age (n = 179) b	0.99	0.97–1	0.151	1	0.99–1.01	0.890
Sex (n = 179)						
Male	1			1		
Female	0.99	0.59–1.66	0.972	1.17	0.83–1.65	0.355
Days of fever prior to visit/admission (n = 162) b	1.22	1.03–1.44	0.020	1.02	0.91–1.13	0.777
Hemoglobin (n = 173) b	0.93	0.93–0.94	<0.001	0.93	0.92–0.94	<0.001
Severe malnutrition (n = 159)						
No	1			1		
Yes	2.08	0.83–5.20	0.115	1.08	0.58–2	0.815
Lactic acidosis (n = 67)						
No	1			NA c		
Yes	0.87	0.37–2.07	0.754	NA	NA	NA
Oxygen saturation (n = 67)						
≥94%	1			NA		
90-<94%	2.12	0.71–6.31	0.173	NA	NA	NA
<90%	1.17	0.44–3.09	0.743	NA	NA	NA
HIV (n = 43)						
No	1			NA		
Yes	1.70	0.6–4.83	0.307	NA	NA	NA
Malaria parasites (n = 179) b	1	0.99–1	0.217	NA	NA	NA

a The analysis was done adjusting by age, sex, malnutrition, hemoglobin and days of fever prior to visit/admission.

b Proportional increment per unit. Age in months, hemoglobin in g/dl and malaria parasites in asexual parasites/µl.

c NA: Not applicable.

After adjusting EPO levels by age, sex, malnutrition, hemoglobin and days of fever prior to visit/admission in the multivariate analysis, EPO only remained associated to Hb concentration (n = 149, p<0.001). Identical results were observed when repeating the analysis stratifying SM in SMA, CM and MSM subgroups (data not shown). When evaluating the role of the clinical group in the association between EPO and Hb no interaction was observed (data not shown).

### Hemolysis markers (bilirubin and LDH) and their relation with Hb in the SM and IBI groups

**Figure 3:**
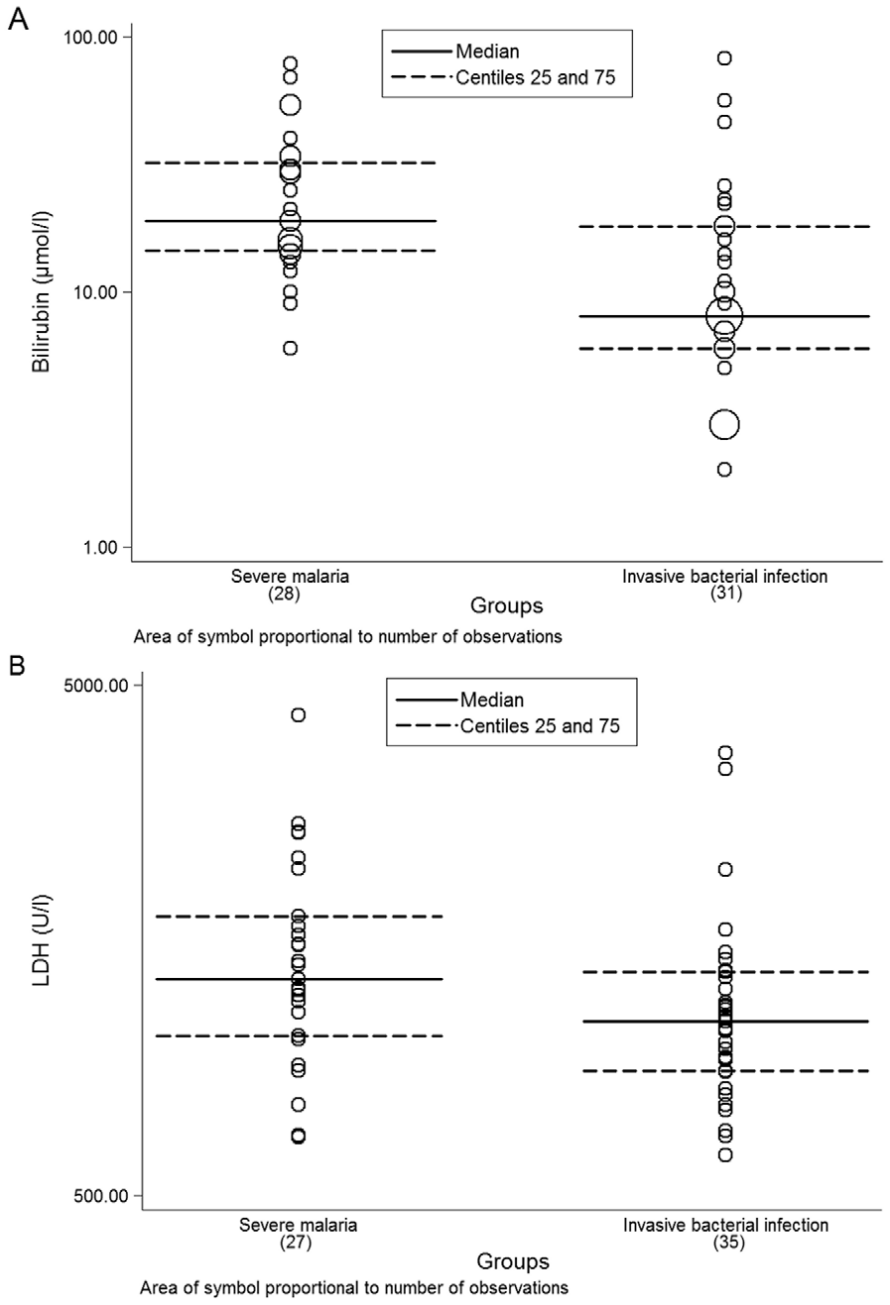
Distribution of bilirubin (µmol/l) and LDH (U/l) in the SM and IBI groups.

As shown in Figure 3A, bilirubin levels in the SM group were higher than in the IBI one (n = 28 and 31, p<0.001). Mean values for bilirubin were 21.62 µmol/l (SD = 1.87 µmol/l) for the SM group and 9.99 µmol/l (SD = 2.4 µmol/l) for the IBI group. When exploring the relation between bilirrubin and Hb, no correlation was observed between the two variables either in the SM and the IBI groups (data not shown).

Although levels of LDH were higher in the SM group compared to the IBI group, values between the two groups overlapped (n = 27 and 35, p = 0.182) (Figure 3B). Mean LDH concentrations were 1564.96 U/l (SD = 800.59 U/l) in the SM group and 1296.83 U/l (SD = 756.90 U/l) in the IBI group. When exploring the relation between LDH and Hb, a negative correlation of values between the two variables was observed in the SM group [β(SE) = −14.75 (7.06), p = 0.047].

## Discussion

In the present study, we have assessed the relation between EPO and malaria-attributable severe disease among children with malaria (uncomplicated, hospitalized without severity and severe) or severe infection (severe malaria and bacteremia). The main findings are that in our setting, despite EPO levels increase according to malaria severity and the SM group presents higher levels of EPO than the IBI group, the utility of EPO to distinguish malaria-attributable severe disease is limited due to the overlap of values between the study groups and the main role of hemoglobin in the expression of EPO.

According to the results, SM cases presented the highest levels of EPO among the study groups and EPO levels increased progressively with malaria severity. Despite being IBI the group with the most severe cases in the study (as reflected by its higher case fatality rate), EPO levels were higher in the SM group when compared to the IBI one. These observations may apparently suggest that EPO could be useful to distinguish malaria-attributable severe disease. However, this conclusion cannot be assumed from this study as the results show that the differences observed on EPO levels depend only on the Hb levels and not on the group (even when considering the different forms of SM separately). In consequence, the visible pattern of EPO distribution among the study groups should vary according to the degree of anemia. This observation contrasts with data from Kenya, where EPO levels were found 3.5 times higher in children with mild malarial anemia than in those without malaria and mild to moderate anemia [27]. A possible explanation to the differences between the two studies could be that the high prevalence (36%) of severe anemia among SM cases in the present study is diluting the effect of other factors linked to the clinical condition in modulating the relation between EPO and Hb. Additionally and irrespectively of the role of Hb in the distribution of EPO, the current overlap of EPO values between the study groups hinders its utility in SM diagnosis.

Although the previous results lack of practicality in terms of improving clinical diagnosis of SM, they can however contribute to understand the pathophysiological processes underlying the expression of EPO. Despite EPO being produced in response to hypoxia and different factors (not only hemolysis) may trigger its expression [24], in our setting hypoxia due to anemia seems to play a principal role as the only factor associated to EPO was Hb. In agreement with that, the highest levels of bilirubin and LDH (both indicators of hemolysis) concentrated in the SM group, coinciding with the highest ratios of severe anemia and EPO levels among the study children. Furthermore, when comparing EPO levels among SMA, CM and MSM, highest values of EPO were found among children with only severe anemia. Also an age-dependent EPO response to anemia has been described in malaria-endemic areas [42], [43], coinciding with the distribution of SMA in younger ages and CM in older ages. However, we could not detect this association in our sample, which may be related to the fact that in Manhiça CM cases also occur among infants and younger children [33]. In our study, 2 out of 6 children with CM were ≤14 months old.

Besides the hypoxia due to anemia, it has been described that in severe malaria the oxygen delivery to tissues is poorer than in sepsis as indicated by the acidosis and the much higher ratios of serum lactate/pyruvate [11], [12]. However, no differences in acidosis and oxygen saturation were detected when comparing SM and IBI groups. This can contribute to explain why no association between EPO levels and study groups was found in our sample. Additionally, the duration of the infection has been associated to the clinical profile of malaria [44], suggesting that this parameter could be related to EPO. No association between duration of the infection (as characterized by number of days of fever prior to visit/admission) and EPO was detected in our sample, but results may be biased as 88% (143/162) of the cases with data of fever duration prior to visit/admission presented the same pattern (≤3 days of fever prior to visit/admission). Why we only observed the effect of Hb in the expression of EPO levels is unclear. A possible explanation is that the strong influence of anemia in our sample may be hiding the contribution of other factors, besides Hb, to the expression of EPO.

Leaving aside the mechanisms that could explain the distribution of EPO levels in this study, the results indicate that the utility of EPO to distinguish malaria-attributable severe disease is limited, at least, in settings with high prevalence of anemia among ill children. From these results, we cannot state that the suspicion of SM on the basis of elevated levels of EPO could be used to accelerate provision of medical assistance in Manhiça. Nevertheless, some studies have suggested the use of biomarkers in Africa for purposes other than clinical diagnosis [45]–[47], whether EPO combined with other parameters could increase the specificity of severe malaria endpoint in epidemiological studies remains to be explored.

In conclusion, malaria parasites can easily be detected with present diagnostic tools but the differentiation of malaria-attributable severe disease remains a challenge. This study suggests that, although EPO levels increase according to malaria severity and are higher in severe malaria than in bacteremia, the utility of EPO to distinguish malaria-attributable severe disease in Manhiça is limited due to the overlap of values between study groups and the main role of hemoglobin in the expression of EPO.
